# Legal Reform to Address Key Drivers of Racial Inequities in Maternal Health: A Multi-method Analysis of California Laws & Regulations from 2019 to 2023

**DOI:** 10.1007/s10995-025-04167-7

**Published:** 2025-11-12

**Authors:** MariaDelSol De Ornelas, Mallory Warner, Linda Jones, Sarah Hooper, Sarah B. Garrett

**Affiliations:** 1 School of Public Health, University of California, Berkeley, USA; 2 University of California College of the Law, San Francisco, USA; 3 California Preterm Birth Initiative, University of California, San Francisco, USA; 4 Philip R. Lee Institute for Health Policy Studies, University of California, San Francisco, 490 Illinois Street, San Francisco, CA 94158 USA; 5 Department of Humanities & Social Sciences, University of California San Francisco, 490 Illinois Street, San Francisco, CA 94158 USA

**Keywords:** Maternal health, Health equity, State policy, Policy analysis

## Abstract

**Objectives:**

California has taken various actions with the goal of advancing maternal health equity. We analyze recent California laws and regulations using a novel conceptual model to understand whether and how they target expert-identified drivers of inequities in maternal health.

**Methods:**

Using policy review and deductive thematic analysis, we evaluated whether recent state laws and regulations in California sought to directly intervene on healthcare-based drivers of racial inequities in maternal health as conceptualized by a CDC-convened expert workgroup.

**Results:**

We identified 13 laws/regulations enacted between 2019-2023 that aimed to improve maternal health. All intervened on one or more healthcare-based drivers of inequities. Two (15%) targeted *Driver 1* – Problems in communication, stereotyping, and other interpersonal interactions, resulting from interpersonal racism, by e.g., requiring provider anti-bias training. One (8%) targeted *Driver 2* – Differential and/or suboptimal treatment for minoritized populations within healthcare settings (e.g., lower-quality care, inequitable burdens of hospital policies; resulting from institutional racism), by making reporting discrimination easier for patients. Twelve (92%) targeted *Driver 3* – Lack of resources and/or policies that could support the health and healthcare of minoritized populations, stemming from structural racism, by e.g., expanding access to midwifery and doula care or diversifying the maternal health workforce.

**Discussion:**

California’s recent maternal health-focused laws/regulations have primarily targeted inadequate or inequitable structural resources *(Driver 3)*. Few directly intervened on *Drivers 1* or *2*. These findings provide a useful grounding for future policy research and reveal the advantages of assessing policies in terms of mechanism-focused intervention targets. Policy implications and potential levers are discussed.

**Supplementary Information:**

The online version contains supplementary material available at 10.1007/s10995-025-04167-7.

## Significance

What is already known about the topic

What this article adds to the literature

A small body of scholarship has mapped the intervention focus of maternal health legislation to levels of racism or other broad descriptive frameworks.

We use a novel conceptual model of the specific manifestations of racism in US maternal healthcare to determine whether California’s recently-enacted laws and regulations seek to intervene on key drivers of racial inequities in maternal health. We find that these policies target healthcare-based drivers of maternal health inequities unevenly, and – by interrogating specific mechanisms – we reveal gaps in both policy and scholarly attention.

**Supplementary Information:**

The online version contains supplementary material available at 10.1007/s10995-025-04167-7.

## Introduction

Racial and ethnic inequities in maternal mortality and severe maternal morbidity (SMM) are a public health crisis in the United States. Non-Hispanic Black and American Indian/Alaska Native (AI/AN, Indigenous) individuals are 3-4 times more likely to die and more likely to suffer from pregnancy-related complications than their white counterparts (Centers for Disease Control and Prevention, 2024; Liese et al., 2019). Maternal health inequities are not explained by individual-level factors (Liese et al., 2019) and a vast literature indicates racism as a root cause of health inequities (Williams et al., 2019). Moreover, most deaths due to pregnancy-related complications are preventable (Trost et al., 2024), suggesting that improvements and interventions targeting racism and its manifestations could substantially reduce maternal health inequities.

In recent years, federal and state policymakers have taken various actions to advance maternal health equity in the United States. A small body of scholarship maps maternal health legislation to well-regarded conceptual models. A systematic policy review of federal and Massachusetts state legislative data between 2010 and 2020, for example, assessed how proposed Black maternal health-related legislation compared on the national and state levels and mapped the bills to Jones’ theorized levels of racism (internalized; interpersonal; and institutional) (Jones, 2000; Carvalho et al., 2021). Similarly, a policy analysis of proposed federal legislation between 2017 and 2021 assessed approaches to reduce racial disparities in pregnancy-related deaths and mapped the legislation to the levels of an ecological framework (Carty et al., 2022). Both Carty et al. and Carvalho et al. used conceptual models that allowed them to detect the levels or domains on which lawmakers focused legislation.

In 2022, a group of maternal health equity experts convened by the U.S. Centers for Disease Control and Prevention (CDC) Foundation—the MMRIA Racism & Discrimination (“MMRIA-RD”) workgroup—authored a conceptual model that included a focus on the mechanisms that underlie maternal health inequities (Hardeman et al., 2022). The experts included Jones (2000), the author of the widely- referenced conceptual model of racism and its effects on health, which Carvalho et al. (2021) engaged. The MMRIA-RD workgroup drew on Jones’ model, a literature review, and workgroup expertise to develop a model that depicted “how racism operates to cause racial inequities in maternal morbidity and severe maternal mortality” (Hardeman et al., 2022). It represented the process by which the historical context of racism has led to contemporary multi-level forms of racism (interpersonal; internalized; institutional; and structural) and illustrated how these forms manifest. The authors defined and illustrated three types of manifestations of racism in healthcare, specifically, that produce maternal health inequities: (1) communication failures and stereotyping by providers; (2) differential treatment of patients; (3) lack of resources and policies that could mitigate inequities. We identify the MMRIA-RD workgroup model as uniquely well-suited to guide an analysis of maternal health policy because it is both tailored to the socio-historical context of US maternal health and because it centers the drivers and mechanisms underlying inequitable outcomes—features that distinguish it from other models linking racism to health.

Drawing on the MMRIA-RD workgroup's model (Hardeman et al., 2022), we conducted an investigation into whether and how California’s recent maternal health legislative and regulatory activities target specific drivers of maternal health inequities. We focus on California because it is a leader in maternal health policy and is the state with the highest number of US births (National Center for Health Statistics, Centers for Disease Control and Prevention, 2024). By mapping California’s policy strategy to known drivers of racial inequities in maternal health, this analysis can reveal its focus and highlight opportunities for legislative and regulatory reform.

## Methods

Our interdisciplinary team used multiple methods across three distinct research activities.

### Policy Review

A legal scholar co-author conducted a comprehensive review of California’s maternal health legislative and regulatory activity enacted between 2019 and 2023. We focused on this 5-year period because it was recent enough to provide timely insights for current policy and practice; feasible in scope to analyze; and long enough to capture a period of increasing attention to health equity. We reviewed both new laws (passed by the California legislature) and new regulations (issued by a California administrative agency to delineate the provisions of policy implementation and enforcement) in order to explore a range of state-level maternal health policy.

The legal scholar reviewed public databases to identify laws and regulations that sought to improve maternal health outcomes and/or maternal health care in California. Search terms and sources were guided by interdisciplinary experts, including the legal scholar. All steps were designed to ensure a comprehensive review. Identified laws and regulations were summarized and provided to coauthors and health policy experts for further review of relevance, accuracy, and completeness. One regulation was removed from consideration as duplicative. We used the remaining laws and regulations in our deductive thematic analysis. See Supplements A and B for full details of our policy review activities and criteria.

### Defining Drivers of Racial Inequities in Maternal Health for Use in Thematic Analysis

In preparation for deductive thematic analysis, we developed definitions, descriptions, and inclusion/exclusion criteria for the three types of “manifestations of racism in healthcare” conceptualized by the MMRIA-RD workgroup (Hardeman et al. 2022). Below we refer to these as “drivers” of inequities in maternal health. We drew on information in Hardeman et al. (e.g., labels, examples, and descriptions) to generate well-described definitions, analytic criteria, and informative titles: Driver 1: Problems in communication, stereotyping, and other interpersonal interactions; Driver 2: Differential and/or suboptimal treatment for racial minorities within healthcare settings; and Driver 3: Lack of resources and/or policies that could support the health and healthcare of racial minority patients receiving maternal healthcare. Though the concepts of each driver are closely related, we sought to develop each driver’s definition and inclusion/exclusion criteria to clearly and consistently distinguish between them in analysis. We practiced applying draft criteria to a subset of laws and, across iterative rounds, further refined and finalized our definitions and inclusion/exclusion criteria for use in thematic analysis. See Table 1 for each Driver’s definition and Supplement C for additional detail.

**Table 1:** Defining the MMRIA-RD workgroup's manifestations of contemporary racism in healthcare for use in deductive thematic analysis

Labels and examples of contemporary racism’s “Manifestations in Healthcare” from the MMRIA-RD workgroup (Hardeman et al., 2022; Figure 1)	Definition of manifestations (“Drivers”) for use in thematic analysis
“Communication failures/Stereotyping by providers - Failed shared-decision making - Concerns not addressed - Delays in treatment - Lack of appropriate referral or consultation - Low expectations of patient engagement - Victim blaming - Ignored and improperly treated symptoms - Ignored and improperly treated pain”	**Driver 1**: Problems in communication, stereotyping, and other interpersonal interactions. **Definition**: This domain concerns the level of individual provider-patient interactions, as well as provider perceptions of and, behaviors toward individual patients. It is related to and stems from interpersonal racism and bias including e.g., implicit bias, microaggressions, stereotyping, and/or the communication failures that can result from these. **Inclusion examples**: Interventions designed to change (improve) how clinicians and/or staff interact with and/or perceive pregnant, birthing, and/or postpartum patients. **Exclusion examples**: Though these may affect interpersonal factors downstream, interventions that aim to change institution- or system-level phenomena would not be coded here (e.g., data reporting requirements; improvements in discrimination-reporting).
“Differential Treatment - Lack of quality care for racial minorities - Restrictions on social support allowed in delivery rooms - Delays in Treatment - Lack of coordinated care - Failure to use a reproductive justice lens or framework in care delivery - Failure to mandate anti-racism training - Failure to offer culturally congruent care”	**Driver 2**: Differential and/or suboptimal treatment for racial minorities within healthcare settings **Definition**: This domain concerns phenomena occurring within health system settings (e.g., clinics, hospitals) regarding differences that can produce inferior and inequitable outcomes and/or experiences for minoritized populations. Interventions may target the lack of supports/features that could reduce these inequities (e.g., lack of — or non-enforcement of — clinician/staff antiracism training; lack of reproductive justice orientation); inferior care for minoritized patients (e.g., inequitable delays in care; lack of culturally congruent care); and policies/procedures that inequitably harm (penalize, inconvenience, alienate, ignore the needs or cultural practices of) minoritized patients, such as restrictions on social support allowed in delivery rooms. **Inclusion examples**: Interventions could seek to mitigate the problems described above by incentivizing or affirmatively requiring something (e.g., improved quality of care for minoritized patients or in primarily minority-serving institutions; universal postpartum depression screening in the context of inequitable screening rates) or by disincentivizing them (e.g., by making it easier for patients to report discrimination). A hallmark of this category is mitigating inequitable care processes and care quality within healthcare settings. Background knowledge of inequities in this realm may be useful to identify relevant policies. **Exclusion examples**: Interventions whose primary focus is creating change in healthcare settings/care processes with no clear equity implication (e.g., incentives to improve overall quality of care in the state). Interventions that primarily and most directly create new resources or policies that may downstream mitigate differential treatment (e.g., the diversification of the state’s maternal-health workforce) but which do not aim to directly intervene on differential/suboptimal treatment.
“Lack of resources/policies - Inadequate staffing in hospitals in majority black neighborhoods - Inadequate hospitals in majority black neighborhoods - Inadequate transportation to prenatal appointments - Lack of providers accepting patients insured by Medicaid - Inadequate resources to support quality infrastructure - Inadequate health insurance coverage and duration - Lack of institutional policy on anti-racism”	**Driver 3**: Lack of resources and/or policies that could support the health and healthcare of racial minority patients receiving maternal healthcare. **Definition**: This domain primarily concerns structural phenomena that occur on levels broader than just the healthcare facility or settings. These phenomena are related to (are manifestations of, sustain and/or could help to reduce) structural racism: “the systems of power based on historical injustices and contemporary social factors that systematically disadvantage people of color and advantage white people through inequities in housing, education, employment, earnings, benefits, credit, media, health care, criminal justice, etc.” (Hardeman et al definition, adapted from Bailey et al. 2017). **Inclusion examples**: Policies that create new resources or policies that could support the health of minoritized populations receiving maternal healthcare. Include supports on the community, state or healthcare system level (e.g., improved perinatal mental healthcare; community health-workers; doulas); or those that create new resources across healthcare systems and/or government programs (e.g., supporting continuity of care as patients transition across healthcare agencies or payers). Include creation of new data-based resources for use at state or health system-level e.g., new data sources or new data-reporting requirements. (Note - not listed in Hardeman et al., but public access to inequities data can both be used by minoritized patients to select hospitals and can be used to incentivize healthcare systems to improve care. Because the latter is about creating new resources/policy, rather than intervening directly on differential treatment, we are coding these in Driver #3). Interventions may explicitly aim to benefit minoritized populations directly or may target a problem disproportionately borne by minoritized populations. **Exclusion examples**: New policies/resources that are not relevant to maternal health and/or that do not propose a policy or resource that would be clearly supportive of minoritized populations, either by targeting their needs directly or by targeting a problem disproportionately borne by minoritized populations. The designation of a day of observance for a maternal health cause, for example, would fall outside of this category if it does not itself provide policies or resources to support the health of minoritized patients receiving maternal healthcare.

### Deductive Thematic Analysis

Two co-authors used the criteria described above to conduct a deductive thematic analysis (Proudfoot, 2023) of the identified laws and regulations. They independently reviewed materials about each law or regulation to evaluate whether it sought to intervene directly on one or more of the focal drivers of racial inequities in maternal health. They categorized the law or regulation as targeting a given driver when the criteria were fulfilled. The coauthors used an Excel database to systematically capture all analyst notes and categorizations. They further honed definitions and categorizations in iterative rounds of independent analysis and discussion. Disagreements were few; all were fully resolved via discussion. A third coauthor reviewed and affirmed the thematic categorizations.

## Results

Of the laws and regulations reviewed, we identified 9 laws and 4 regulations (*N* = 13) enacted in California between January 1, 2019, and December 31, 2023, that represented distinct efforts to improve maternal health (Table 2). All four regulations were developed by California’s Department of Health Care Services as part of California Advancing and Innovating Medi-Cal (Cal-AIM), the state’s effort to transform its Medicaid program (Department of Health Care Services, 2022). One law that had multiple distinct requirements — SB464 (California Dignity in Pregnancy and Childbirth Act, 2019) — targeted all three drivers. Its requirements are described separately in each section below.

**Table 2:** California laws and regulations mapped to drivers of maternal health inequities, by year (2019–2023)

Year enacted	Name of law or regulation	Purpose	Provisions/requirements	Driver #1	Driver #2	Driver #3	Law (*n*=)	Regulation (*n*=)
2019	California Dignity in Pregnancy and Childbirth Act (SB 464)	To decrease implicit bias by clinicians; ensure patients know their rights and how to report discrimination; and improve the way pregnancy-related death data is collected and shared.	Implicit bias trainings must be provided by hospitals and alternative birth centers for perinatal providers every 2 years. The training must include 10 topics including implicit bias, its effects, and steps to decrease it at individual and institutional levels, as well as topics such as health inequities in perinatal care, communication, reproductive justice, and the effects of the “historical and contemporary exclusion and oppression of minority communities.”	X			1	
			Patient education and information must be provided by hospitals to patients, upon admission, on the right for patients to be free of discrimination and how to file a report with state-level entities (California Department of Health, California Civil Rights Department, Medical Board of California) if patients experience discrimination during health care delivery.		X			
			Expand data on death certificates to include additional information about timing of pregnancy-related deaths and the California Department of Public Health must track and publicly share information about pregnancy-related deaths, by race, ethnicity, and region.			X		
2019	Health care coverage: maternal mental health (AB 577)	To ensure that women with maternal mental health conditions have continued access to necessary care (up to 12 months postpartum), even if their provider’s contract with their health plan ends or they change health plans.	Health insurance plans must extend coverage for maternal mental health conditions for a maximum of 12 months and under specific circumstances, the bill ensures that patients can complete their treatment with their original healthcare provider.”			X	1	
2019	Continuing education: physicians and surgeons: maternal mental health (AB 845)	To enhance physicians’ and surgeons’ knowledge and competence in maternal mental health by incorporating specific topics, such as cultural competency and implicit bias, into continuing education requirements.	The Medical Board of California must consider including a course on maternal mental health in continuing education requirements for physicians and surgeons. The course should include specific topics such as cultural competency and implicit bias.	X			1	
2020	Nurse-midwives: scope of practice (AB 1237)	To allow CNMs in California to practice more independently while establishing new requirements for oversight and data collection.	Expands the scope of practice for CNMs allowing them to attend low-risk pregnancies and childbirths without physician supervision.			X	1	
2021	Maternal care and services (SB 65)	To improve maternal and infant health outcomes, with a specific focus on addressing racial disparities.	California Pregnancy-Associated Mortality Review Committee (CA-PAMR) includes mandates that expand and codifies the CA-PAMR to investigate maternal mortality and morbidity, examine racial and socioeconomic disparities in maternal health outcomes, assess birthing outcomes for queer, transgender, and gender non-conforming individuals, and develop and propose best practices to reduce maternal and infant mortality and morbidity rates			X	1	
			Midwifery Workforce Training Act establishes a program under the Office of Statewide Health Planning and Development to contract with programs that train CNMs and licensed midwives and requires the office to contract only with programs that include or intend to include training components designed for medically underserved multicultural communities, lower socioeconomic neighborhoods, or rural communities			X		
			Medi-Cal Doula Benefit provides eligible pregnant and postpartum Medi-Cal members with comprehensive doula services, including prenatal and postpartum visits and labor support, with specific reimbursement rates and provider requirements, although utilization has been limited so far.			X		
2021	CalAIM initiatives (regulations)							
	Medi-Cal Postpartum Expansion	To expand postpartum coverage to all medically necessary services during the 12-month postpartum period for Medi-Cal eligible patients.	Extends the postpartum coverage period from 60 days to 12 months (365 days) after the end of pregnancy and includes coverage for mental health services without requiring a mental health diagnosis			X		1
	Community Health Workers Preventive Services	To provide preventive health services to eligible Medi-Cal members to prevent disease, disability, and other health conditions including perinatal health conditions.	Preventive Services are provided by Community Health Workers that provide eligible members with health education, screening/assessments of health-related social needs, health navigation for community resources, and patient advocacy for community support.			X		1
	Enhanced Care Management and Community Support	To provide comprehensive care coordination and optional services for Medi-Cal members with complex health needs, including Pregnant and Postpartum Population of Focus.	Comprehensive care coordination and optional services for the Pregnant and Postpartum Population of Focus (PoF) expands to include ‘birth equity’ in their PoF definition to address known disparities in health and birth outcomes in racial and ethnic groups with high maternal morbidity and mortality rates.			X		1
	Equity and Practice Transformation Payments Program	To provide funding for practice transformation activities aimed at advancing health equity and population health in primary care settings.	Allocates $700 million over five years (2023–2027) towards primary care practices providing pediatric, family medicine, internal medicine, or OB/GYN services to Medi-Cal members that aim to implement practices to advancing health equity and improving population health.			X		1
2021	Hospital equity reporting (AB 1204)	To increase transparency, identify health disparities, and promote actions to address inequities in hospital care across California.	Hospitals must prepare an annual equity report that analyzes health status and access to care disparities for patients based on age, sex, race, ethnicity, language, disability status, sexual orientation, gender identity, and payor.			X	1	
2022	Health care coverage: maternal and pandemic-related mental health conditions (SB 1207)	To enhance health care coverage for maternal mental health conditions	Encourages health plans and insurers to improve screening, treatment, and referral to maternal mental health services and include coverage for doulas and training for obstetric providers.			X	1	
2023	Health care coverage: doulas (AB 904)	To increase access to doula services and integrating them into existing health care coverage frameworks.	Requires health care service plans and health insurers to develop and implement doula programs that address racial health disparities in maternal and infant health outcomes.			X	1	
2023	Healing arts: Pregnancy and childbirth (SB 667)	To expand access to healthcare for pregnant individuals by removing barriers for CNMs to practice to the full extent of their training and scope.	Allows CNMs to attend cases of low-risk pregnancy and childbirth, clarifies that CNMs do not need to be “in the same practice with” a physician to consult and collaborate, and clarifies their ability to dispense medication directly to patients.			X	1	
Subtotals							9	4
Total laws and regulations							13	

*Abbreviations: *CalAIM *California Advancing and Innovating Medi-Cal, *CNMs *Certified Nurse-Midwives, *Medi-Cal *California Medical Assistance Program, *OB/GYN* Obstetrician-gynecologist

### Driver 1

Two laws (15%) targeted Driver 1—problems in communication, stereotyping, and other interpersonal interactions. California Dignity in Pregnancy and Childbirth Act (2019) mandated that all California hospitals, alternative birth centers, and primary care clinics offering alternative birth center services provide implicit bias training for perinatal providers every 2 years, and that the providers participate in that training. The law outlined 10 required training topics (Table 2). The second law (Continuing Medical Education: Physicians and Surgeons, 2020) required that the Medical Board of California consider including a course on maternal mental health in continuing education requirements for physicians and surgeons, with specific topics including cultural competency and implicit bias.

### Driver 2

One law (8%) targeted Driver 2—differential and/or suboptimal treatment for racial minorities within healthcare settings. It concerned a different component of the California Dignity in Pregnancy and Childbirth Act (2019), which required that hospitals provide patients, upon admission, with written information about their rights to be free from discrimination and information about how to file a report with specific state-level entities if they experience discrimination. Previously, hospitals had not been required to provide such information to patients.

### Driver 3

Twelve laws and regulations (92%) targeted Driver 3—lack of resources and/or policies that could support the health and healthcare of racial minorities. Their provisions and requirements spanned four areas. One area was expanding coverage for and access to maternal health services, including doula care. A second area focused on expanding the scope of practice for certified nurse-midwives, which allows midwives to care for a broader range of patients, including those with more complex conditions. Greater access to midwifery and doula services was the modal focus of Driver 3-focused legal reform (5 of 12 laws or regulations). A third area concerned increasing care coordination and preventive services for underserved perinatal populations, including screening and referrals for health-related social needs. The fourth area was improving data collection and reporting related to maternal race and ethnicity. These laws had the goal of revealing and measuring inequities to inform change (e.g., in guiding quality improvement or the allocation of resources).

## Discussion

Our findings show that California’s recent maternal health-focused laws and regulations (enacted 2019–2023) targeted key drivers of maternal health inequities. They primarily targeted inadequate or inequitable structural resources (Driver 3). Few directly addressed problems in providers’ interactions with patients (e.g., stereotyping), inequities in patient treatment, or discrimination in health care delivery, which are represented by Drivers 1 and 2. This analysis presents a novel analytic approach and contemporary insights that scholars and advocates can use to understand California’s policy strategy.

Our findings contribute to a small body of scholarship on maternal health policy objectives. Carvalho et al.’s (2021) systematic policy review of federal and Massachusetts state legislative data from 2010 to 2020 revealed increases in proposed legislation that sought to address racial disparities and equity in maternal health. Four of the 47 introduced bills they studied became law (two federal, two state), all of which the authors categorized as addressing institutional racism. These laws — like nearly all of the proposed legislation — targeted upstream factors such as expanding access to healthcare, improving health data collection, and supporting Maternal Mortality Review Committees. Similarly, Carty et al. (2022) analyzed federal maternal health legislation introduced from 2017 to 2021 and also identified a major focus on upstream factors. They located the most common approaches at what they labeled the organizational/institutional level (e.g., healthcare-related approaches such as care coordination; workforce diversification; enhancing data and maternal mortality review) followed by approaches on the community/societal level (e.g., Medicaid expansion; programs to address social determinants of health). The latter included a focus on expanding Medicaid coverage for care provided by doulas and midwives—also a major focus that we detected in California’s legal reform. Of 37 federal bills introduced, Carty et al. found that only two became law, both of which included approaches at the organizational/institutional level (e.g., enhancing data and maternal mortality review; maternal care coordination and training). Though the scope and conceptual frameworks of our study and those of Carvalho et al. and Carty et al. differ, certain findings align: recent federal, Massachusetts, and California maternal health legislation and laws primarily address factors upstream from maternal health (i.e., institutional and structural factors) and upstream from specific manifestations of racism in healthcare delivery. In a departure from the other studies, however, our study additionally identified state laws that directly target problems in communication and stereotyping (Driver 1) or differential/suboptimal treatment for racial minorities (Driver 2) — two of the three theorized manifestations of racism in maternal healthcare (Hardeman et al., 2022).

Our findings have both methodological and real-world implications. Regarding the former, our findings reveal the strengths of using a model, like the MMRIA-RD workgroup's (Hardeman et al., 2022), that focuses on the mechanisms by which racism operates to produce inequities in U.S. maternal mortality and morbidity. Categorizing policies into broad levels or domains, such as ‘institutional racism” or “organizational/institutional,” can reveal that there is legal reform focused on addressing certain upstream factors or qualities of healthcare institutions. In contrast, using a domain-specific and mechanism-focused lens, such as the MMRIA-RD workgroup‘s model, revealed even more about the policies under study. By deductively investigating whether legal reform targeted inequitable treatment within healthcare institutions, for example, we discovered a paucity of California laws and regulations in this space (Driver 2). The model enabled us to center our inquiry on specific and actionable inequity-producing mechanisms in domestic maternal health. Such a conceptual lens could also be useful for future research that seeks to assess the effectiveness of legislation for intended outcomes.

Beyond academia, our findings can support the development of strategic targets for advocates and policymakers. The substantial focus of legislation targeting Driver 3 is encouraging, reflecting California’s marked prioritization of social determinants of health since 2016 (Tong & Elizabeth, 2022) and the importance of undoing structural racism (Hardeman et al., 2022), as well as the breadth of the category’s scope. It will be productive for future analyses to assess the quality, implementation, and impact of Driver 3-directed efforts on maternal health outcomes. Only two laws addressed Driver 1, but one of them—heralded as an historic effort to reduce bias and racism in maternal health care (California Dignity in Pregnancy and Childbirth Act, 2019) — aimed to mitigate many of the interpersonal communication problems identified by the MMRIA-RD workgroup.

Laws and regulations targeting Drivers 1 and 3 may eventually influence the quality and equity of healthcare provided (Driver 2), but we identified only one law that directly addressed it (California Dignity in Pregnancy and Childbirth Act, 2019). Moreover, the component of that law that addresses Driver 2—though important — is quite narrowly focused. Most example manifestations of racism that the MMRIA-RD workgroup (Hardeman et al., 2022) included in the “differential treatment” category are unaddressed by California laws or regulations in the 5-year period. The limited attention in this domain is concerning. Differential and suboptimal treatment of minority populations within healthcare systems is increasingly addressed in maternal health quality improvement work led by independent collaboratives or healthcare systems themselves. However, communities have long expressed that healthcare systems do not hold themselves adequately accountable for equitable and quality care (Cummings, 2022; White VanGompel et al., 2022), and a lack of robust data on inequitable care processes can inhibit these efforts (McPherson & De Maio, 2023). Moreover, Civil Rights law to discourage and penalize discrimination in healthcare (Civil Rights Act of 1964, Title VI) has had modest effects. This has been due to both challenging evidentiary and reporting requirements (Chandra et al., 2017) and to its ineffectiveness to address more subtle and unconscious manifestations of racism, which by the end of the 20th century had largely replaced problems of “blatant racism and discrimination” in healthcare (Matthew, 2018).

Given the limited attention to Driver 2 in recent maternal health policy and the harms stemming from inequitable care processes (Hardeman et al., 2022), advocates and policymakers should consider prioritizing laws and regulations with direct interventional impact. Adapting approaches from other settings may be a productive step towards this goal. Considering Medicaid policy, eleven state programs have required maternal/caregiver depression screenings to be done during well-child visits (National Academy for State Health Policy, 2023) – introducing a universal requirement to address disparate screening rates. In the area of organ transplant, a federally-established governing board adjusted organ transplant waiting times for patients disadvantaged by previous race-based measures (United Network for Organ Sharing, 2022). State policymakers could additionally build on examples from historic state Medicaid programs (Department of Health Care Services, 2020), new accreditation requirements (The Joint Commission, 2022), or new federal opportunities, such as the “Transforming Maternal Health Model” (Centers for Medicare & Medicaid Services, 2023). More immediately, in the absence of policy mandates, healthcare systems can work to hold themselves accountable to equity change through a variety of means. Some contemporary examples include linking equity metrics to compensation of physician groups or executives (Rodriguez et al., 2024; Marill, 2022); focusing quality improvement and measurement efforts on care process inequities (Andrews, 2023; Cummings, 2022); or developing community-hospital partnerships with accountability mechanisms (Marshall et al., 2025).

We note that our findings are limited to the language of enacted laws and regulations. They do not reflect the focus or impact of pre-existing policies, nor do they reflect the implementation of the focal laws and regulations. Even well-targeted reforms can be hobbled by post-enactment changes (e.g., budget reductions) or implementation challenges, which may reduce their potential effects. For example, greater access to midwives and doulas — interventions that experts and advocates identify as key ways to improve maternal health equity (Altman et al., 2020; The White House, 2022)—was the focus of approximately half of the laws and regulations we identified as targeting Driver 3. However, varied challenges have hindered efforts to increase patient access to doulas and midwifery care (Marshall et al., 2024; Thompson-Lastad et al., 2024). The major policy targeting Driver 1 (California Dignity in Pregnancy and Childbirth Act, 2019) was similarly threatened by implementation challenges, lacking implementation support and enforcement mechanisms (Montague et al., 2023) and yielding low compliance rates (California Department of Justice, 2023). Solutions to these implementation problems have been identified by scholarly, patient, and birth-worker communities (Garrett et al., 2023; Marshall et al., 2024; Thompson-Lastad, 2024). To fully understand how California’s maternal health policies impact maternal health, researchers must complement this analysis of policy strategy with an assessment of policy quality and implementation as well. Attention to Medicaid policies that exist on the state and federal levels is similarly crucial for a comprehensive picture of the maternal health policy landscape.

## Conclusions

Between 2019 and 2023, California’s maternal health-focused laws and regulations primarily targeted inadequate resources and/or structural policies that contributed to racial inequities (Driver 3). Few laws or regulations sought to directly intervene on problems in providers’ interactions with patients (e.g., stereotyping; Driver 1) or on inequitable or discriminatory health care delivery (Driver 2). We urge policymakers and advocates to consider policy levers that can directly and durably mitigate all three healthcare-based drivers of maternal health inequities identified by the MMRIA-RD workgroup (Hardeman et al.,^ 2022^).

Mapping laws and regulations to a conceptual model that is maternal health-specific and focused on the mechanisms through which racism operates is a productive approach to understanding maternal health policy strategy. Though our focus was on the language of enacted laws and regulations in California, this analytic approach may be applied to other state or federal policies and could structure inquiry into policy impacts on health outcomes. Researchers may additionally explore its utility for policy analysis concerning other clinical settings with pronounced health inequities. We aim to extend this analysis to past state legislation and to the national context to more fully understand maternal health policy interventions, their implementation, and their effects on long-standing maternal health inequities.

## Supplementary Information

Below is the link to the electronic supplementary material.

Supplementary Material 1

## Data Availability

Focal data are within the Tables and Supporting Information files. Researchers may contact the corresponding author for additional supporting data and methodological detail (e.g., policy summaries, full listing of landscape review sources).

## References

[CR1] Altman, M. R., McLemore, M. R., Oseguera, T., Lyndon, A., & Franck, L. S. (2020). Listening to women: Recommendations from women of color to improve experiences in pregnancy and birth care. *Journal of Midwifery & Women’s Health,**65*(4), 466–473. 10.1111/jmwh.13102 10.1111/jmwh.13102 32558179

[CR2] Andrews, C. (2023). Children’s & Women’s Hospital launches program to improve maternal health equity. https://www.usahealthsystem.com/news/cw-launches-program-improve-maternal-health-equity

[CR36] Bailey, Z. D., Krieger, N., Agéénor, M., Graves, J., Linos, N., & Bassett, M. T.(2017). Structural racism and health inequities in the USA: Evidence and interventions. The Lancet, 389(10077), 1453–1463. 10.1016/S0140-6736(17)30569-X 28402827

[CR3] California Dignity in Pregnancy and Childbirth Act, 123630-123630.7 Health and Safety Code § Article 4.6 (2019). Added by Stats. 2019, Ch. 533, Sect. 3 https://leginfo.legislature.ca.gov/faces/codes_displayText.xhtml?lawCode=HSC&division=106.&title=∂=2.&chapter=2.&article=4.6

[CR4] California Department of Justice (2023). Report on Healthcare Facilities and the California Dignity in Pregnancy and Childbirth Act https://oag.ca.gov/system/files/attachments/press-docs/Report%20on%20Healthcare%20Facilities%20and%20the%20California%20Dignity%20in%20Pregnancy%20and%20Childbirth%20Act%20%282%29.pdf

[CR5] Carty, D. C., Mpofu, J. J., Kress, A. C., Robinson, D., & Miller, S. A. (2022). Addressing racial disparities in pregnancy-related deaths: An analysis of maternal mortality-related federal legislation, 2017–2021. *Journal of Women’s Health,**31*(9), 1222–1231. 10.1089/jwh.2022.0336 36112423 10.1089/jwh.2022.0336 PMC10949966

[CR6] Carvalho, K., Kheyfets, A., Maleki, P., Miller, B., Abouhala, S., Anwar, E., & Amutah-Onukagha, N. (2021). A systematic policy review of Black maternal health-related policies proposed federally and in Massachusetts: 2010–2020. *Frontiers in Public Health,**9*, Article 664659. 10.3389/fpubh.2021.664659 34746071 10.3389/fpubh.2021.664659 PMC8566737

[CR7] Centers for Medicare & Medicaid Services (2023). Transforming Maternal Health (TMaH) Model https://www.cms.gov/priorities/innovation/innovation-models/transforming-maternal-health-tmah-model

[CR8] Centers for Disease Control and Prevention (2024). Pregnancy Mortality Surveillance System. Maternal Mortality Prevention https://www.cdc.gov/maternal-mortality/php/pregnancy-mortality-surveillance/index.html

[CR9] Chandra, A., Frakes, M., & Malani, A. (2017). Challenges to reducing discrimination and health inequity through existing civil rights laws. *Health Affairs (Project Hope)*, *36*(6), 1041. 10.1377/hlthaff.2016.1091 28583962 10.1377/hlthaff.2016.1091 PMC5654529

[CR37] Civil Rights Act of 1964, Pub. L. No. 88-352, 78Stat. 241 (1964). https://www.govinfo.gov/app/details/STATUTE-78/STATUTE-78-Pg241

[CR10] Continuing Medical Education: Physicians and Surgeons: Maternal Mental Health, 2190-2196.9 Business and Professions Code § Article 10 (2020). Added by Stats. 2019, Ch. 220, Sect. 1 https://leginfo.legislature.ca.gov/faces/codes_displaySection.xhtml?lawCode=BPC&sectionNum=2196.9.&article=10.&highlight=true&keyword=maternal%20mental%20health

[CR11] Cummings, L. (2022). Listening to Black Californians: How the Health Care System Undermines Their Pursuit of Good Health https://www.chcf.org/wp-content/uploads/2022/09/ListeningBlackCalifornians.pdf

[CR12] Department of Health Care Services (2020). P​​​​​​​​​​ublic Hospital Redesign & Incentives in Medi-Cal Program (PRIME) https://www.dhcs.ca.gov/provgovpart/Pages/PRIME.aspx

[CR13] Department of Health Care Services (2022). Medi-Cal Transformation. https://calaim.dhcs.ca.gov/

[CR14] Garrett, S. B., Jones, L., Montague, A., Fa-Yusuf, H., Harris-Taylor, J., Powell, B., Chan, E., Zamarripa, S., Hooper, S., & Butcher, C., B. D (2023). Challenges and opportunities for clinician implicit bias training: Insights from perinatal care stakeholders. *Health Equity*, *7*(1), 506–519. 10.1089/heq.2023.0126 37731787 10.1089/heq.2023.0126 PMC10507933

[CR15] Hardeman, R. R., Kheyfets, A., Mantha, A. B., Cornell, A., Crear-Perry, J., Graves, C., Grobman, W., James-Conterelli, S., Jones, C., Lipscomb, B., Ortique, C., Stuebe, A., Welsh, K., & Howell, E. A. (2022). Developing tools to report racism in maternal health for the CDC maternal mortality review information application (MMRIA): Findings from the MMRIA racism & discrimination working group. *Maternal and Child Health Journal,**26*(4), 661–669. 10.1007/s10995-021-03284-3 34982327 10.1007/s10995-021-03284-3

[CR16] Jones, C. P. (2000). Levels of racism: A theoretic framework and a gardener’s tale. *American Journal of Public Health,**90*(8), 1212–1215. 10936998 10.2105/ajph.90.8.1212 PMC1446334

[CR17] Liese, K. L., Mogos, M., Abboud, S., Decocker, K., Koch, A. R., & Geller, S. E. (2019). Racial and ethnic disparities in severe maternal morbidity in the United States. *Journal of Racial and Ethnic Health Disparities,**6*(4), 790–798. 10.1007/s40615-019-00577-w 30877505 10.1007/s40615-019-00577-w

[CR18] Marill, M. C. (2022). *Raising the stakes to advance equity in black maternal health*. *Health Affairs*. 10.1377/hlthaff.2022.00036 35254937 10.1377/hlthaff.2022.00036

[CR19] Marshall, C., Nguyen, A., Yang, C. E., & Gómez, A. M. (2024). Facilitators and barriers to Medicaid doula benefit implementation in California: Perspectives from managed care plans and risk-bearing organizations. *Women’s Health Issues,**34*(5), 465–472. 10.1016/j.whi.2024.05.006 38971691 10.1016/j.whi.2024.05.006 PMC12186737

[CR20] Marshall, C., Nguyen, A., Cuentos, A., Almenar, A., Mace, G., Arcara, J., Jackson, A. V., & Gómez, A. M. (2025). An interprofessional collaboration between a Community-Based doula organization and clinical partners: The champion dyad initiative. *Journal of Midwifery & Women’s Health*. 10.1111/jmwh.13730 10.1111/jmwh.13730 PMC12172575 39825873

[CR21] Matthew, D. B. (2018). *Just medicine: A cure for racial inequality in American health care*. New York University.

[CR22] McPherson, M., & De Maio, F. (2023). Measuring progress toward equity in health care. *Journal of Health Care for the Poor and Underserved*, *34*(4), 1445–1451. 38661766

[CR23] Montague, A., Garrett, S., & Hooper, S. (2023). *MEND Policy Report: Legal analysis, stakeholder insights, & policy recommendations for healthcare provider implicit vias training in California*. University of California. https://mend.ucsf.edu/document/policy-report-legal-analysis-stakeholder-insights-policy-recommendations-for-healthcare-provider

[CR24] National Academy for State Health Policy (2023). Medicaid Policies for Caregiver and Maternal Depression Screening during Well-Child Visits, by State [Dataset] https://nashp.org/state-tracker/maternal-depression-screening/

[CR25] National Center for Health Statistics, Centers for Disease Control and Prevention (2024). State and Territorial Data [Dataset] https://www.cdc.gov/nchs/fastats/state-and-territorial-data.htm

[CR26] Proudfoot, K. (2023). Inductive/deductive hybrid thematic analysis in mixed methods research. *Journal of Mixed Methods Research,**17*(3), 308. 10.1177/15586898221126816

[CR27] Rodriguez, H. P., Epstein, S. D., Brewster, A. L., Brown, T. T., Chen, S., & Bibi, S. (2024). Launching financial incentives for physician groups to improve equity of care by patient race and ethnicity. *The Milbank Quarterly*, *102*(4), 944–972. 10.1111/1468-0009.12720 39450693 10.1111/1468-0009.12720 PMC11654755

[CR28] The Joint Commission (2022). Maternal Levels of Care Verification https://www.jointcommission.orghttps://www.jointcommission.org/what-we-offer/verification/maternal-levels-of-care-verification/

[CR29] The White House (2022) The White House Blueprint for Addressing the Maternal Health Crisis https://www.whitehouse.gov/wp-content/uploads/2022/06/Maternal-Health-Blueprint.pdf

[CR30] Thompson-Lastad, A. (2024). Medi-Cal Access to Community Midwifery Care: Midwives’ Perspectives https://osher.ucsf.edu/sites/osher.ucsf.edu/files/inline-files/Medi-Cal%20Midwifery%20Policy%20Brief.pdf

[CR31] Tong, M., & Elizabeth, H. (2022). California Efforts to Address Behavioral Health and SDOH: A Look at Whole Person Care Pilots. https://www.kff.org/mental-health/issue-brief/california-efforts-to-address-behavioral-health-and-sdoh-a-look-at-whole-person-care-pilots/

[CR32] Trost, S., Beauregard, F., & Njie, F. (2024). Pregnancy-Related Deaths: Data From Maternal Mortality Review Committees in 36 U.S. States, 2017–2019. CDC https://www.cdc.gov/maternal-mortality/php/data-research/mmrc-2017-2019.html

[CR33] United Network for Organ Sharing (2022). Implementation notice: Requirement for race-neutral eGFR formulas in effect. UNOS https://unos.org/news/implementation-notice-race-neutral-egfr-formulas/

[CR34] White VanGompel, E., Lai, J. S., Davis, D. A., Carlock, F., Camara, T. L., Taylor, B., Clary, C., McCorkle-Jamieson, A. M., McKenzie-Sampson, S., Gay, C., Armijo, A., Lapeyrolerie, L., Singh, L., & Scott, K. A. (2022). Psychometric validation of a patient-reported experience measure of obstetric racism© (The PREM-OB Scale^™^ suite). *Birth*, *49*(3), 514–525. 10.1111/birt.12622 35301757 10.1111/birt.12622 PMC9544169

[CR35] Williams, D. R., Lawrence, J., & Davis, B. (2019). Racism and health: Evidence and needed research. *Annual Review of Public Health,**40*, 105. 10.1146/annurev-publhealth-040218-043750 30601726 10.1146/annurev-publhealth-040218-043750 PMC6532402

