# Supplementary material for: Legal Reform to Address Key Drivers of Racial Inequities in Maternal Health: A Multi-method Analysis of California Laws & Regulations from 2019 to 2023
Source: Matern Child Health J. 2025 Nov 12;30(2):252–62. doi: 10.1007/s10995-025-04167-7 (PMC12999683; doi:10.1007/s10995-025-04167-7)
Supplement: Supplementary file 1 — Supplementary Material 1 [file 10995_2025_4167_MOESM1_ESM.pdf]

Supplemental Materials for "Legal reform to address key drivers of racial inequities in maternal health: A multi-method analysis of California laws & regulations from 2019 to 2023"

Supplement A 2

Supplement B 4

Supplement C 5

Supplement A

Policy Review Process to Identify California Laws and Regulations Enacted 2019 through 2023 that Sought to Improve Maternal Health

**Phases of search and review**	**Review activities and criteria**
A. Identifying relevant laws and regulations	Conducted review on LegiScan website* and California administrative agency websites (California (CA) Department of Health Care Services, California Department of Managed Health Care, Department of Health Care Access and Information) to identify all laws and regulations enacted and issued between 2019 and 2023 that met the following criteria:<br><br>• Keywords/Terms searched included: matern! OR preg! OR birth OR partu! OR midwi! OR "health equity"<br>• Had the explicit goal of directly targeting maternal health outcomes and/or care (prenatal, perinatal, or postpartum) for improvement; or addressed the implementation of a law that itself had the explicit goal of directly targeting maternal health outcomes and/or care (prenatal, perinatal, or postpartum) for improvement.<br>• We included in our consideration of outcomes both clinical outcomes and experienctial outcomes of pregnant, birthing and/or postpartum individuals. The policy targets could be proximate to care delivery (e.g., discrimination in healthcare settings) or could be more distal (e.g., changes to the perinatal workforce or to the data used to understand or reduce the burden of maternal mortality/morbidity).<br>• Exclusion criteria:<br>i. No suggested connection to health, healthcare, health services, health insurance/coverage.<br>ii. Health-related but:<br>• Primary focus was basic science (e.g., medicinal drug development, biomarkers) unrelated to maternal/child health.

	• Primary focus was an aspect of the healthcare system healthcare settings, or health condition unrelated to maternal/child health.<br>• Primary focus was abortion care or access; or contraceptive care or access; or infant/child health only, which were outside of the scope of this study.
B. Documentation of eligible laws and regulations	Documentation, download, and structured summary created of law/regulation text for those identified as satisfying criteria above.
C. Presentation of eligible laws and regulations to policy expert team	Presentation and discussion of eligible laws and regulations with co-authors and health policy experts to evaluate results, accuracy, and completeness of search process.<br><br>• Regulations designed to support the implementation of a law included in our analysis, but which did not themselves represent a distinct approach to improve maternal health outcomes and/or care, were removed from the sample.<br>• Sources reviewed to gain supplemental information about legislative and regulatory activities are reported in Supplement B.<br><br>The resulting laws and regulations were included in the next analytic phase (thematic analysis).
*Settings for LegisScan Website were: State: “California”; Sessions: “2019-2020”; “2021-2022”; “2023-2024”; Type: “Bills”; Status: “Passed.”	

Supplement B

Primary and secondary sources used for review of and supplementary inquiry into legislative and regulatory activities

Primary Sources

- California Department of Health Care Services
  - CalAIM, Medi-Cal Transformation
  - Enhanced Care Management Policy Guide
  - Equity and Practice Transformation Payments Program
  - Approved State Plan Amendments
- California Legislative Information
- LegiScan

Secondary Sources

- Black Women for Wellness Action Project
- California Black Health Network
- California Department of Health Care Access and Information (HCAI)
- California Department of Public Health: Maternal, Child and Adolescent Health Division
- California Governor's Budget Proposal
- California Health Care Foundation
- California Maternal Quality Care Collaborative
- California Medical Association
- California Momnibus Act
- CDC.Gov - Enhancing Reviews and Surveillance to Eliminate Maternal Mortality
- Center for Health Care Strategies
- The Commonwealth Fund
- Department of Managed Health Care - 2022 Health Equity and Quality Committee Recommendations Report
- Health Affairs
- Health Care Transformation Task Force
- Kaiser Family Foundation
  - Medicaid
  - Medicaid Postpartum Extension Tracker
- New York Times
- Policy Center for Maternal Health
- Public Health Institute - California Pregnancy-Associated Mortality Review
- United States Department of Health and Human Services: Healthy People 2030
- Urban Institute
  - Understanding Training and Workforce Pathways to Develop and Retain Black Maternal Health Physicians in California
- Washington Post

Supplement C

Additional methodological notes about the operationalization and adaptation of Hardeman et al categories for use in thematic analysis

This information complements the information presented in Table 1 of the accompanying manuscript.

Driver #1

Driver #1 was clearly distinguishable from Drivers #2 and #3 without elaborating definitional or inclusion/exclusion criteria.

Drivers #2

To delineate the scope of Driver #2 and to clearly distinguish from Driver #3, we specify that Driver #2 concerns phenomena within healthcare settings.

We interpret the biggest difference between Driver #2 and Driver #3 is that #2 pertains to phenomena happening within healthcare settings and directly targets inequities there.

Reflecting on Hardeman et al examples, “failure to mandate antiracism training” and “restrictions on social support” are two examples of healthcare institution-level policies that Hardeman et al listed in their 2nd category— manifestations of racism as “differential treatment” in healthcare. These are aligned with our operationalization of Driver #2. However, Hardeman et al list “lack of institutional policy on anti-racism” in their third category, “lack of resources/policies.” We worked to clarify this example as we operationalized our categories. To the extent that “lack of institutional policy on anti-racism” refers to a problem within a given healthcare institution, we include it in Driver #2. Doing so co-locates it with other healthcare institution-level policies (or lack of policies) that could produce differential care/treatment of minoritized patients (e.g., not mandating antiracism training). However, to the extent that “lack of institutional policy on anti-racism” refers to, for example, the lack of a state requirement for facilities to have an antiracism policy, that aligns with how we have operationalized Driver 3, below.

Unpacking this nuance allowed us to create more conceptually clear categories to use in analysis.

Driver #3

To delineate the scope of Driver #3 and to more clearly distinguish it from Driver #2, we specify in our definition that Driver #3 typically concerns phenomena outside of specific healthcare settings - more focused on community-, system-, or state-level phenomena. Referencing the clarification work above regarding antiracism policy, Driver 3 concerns phenomena that are broader than and/or external to the healthcare institution/facility setting.
